# The effect of age and resilience on the dose–response function between the number of adversity factors and subjective well-being

**DOI:** 10.3389/fpsyg.2024.1332124

**Published:** 2024-02-09

**Authors:** Shulan Hsieh, Yun-Hsuan Chang, Zai-Fu Yao, Meng-Heng Yang, Cheng-Ta Yang

**Affiliations:** ^1^Department of Psychology, National Cheng Kung University, Tainan, Taiwan; ^2^Institute of Allied Health Sciences, National Cheng Kung University, Tainan, Taiwan; ^3^Department of Public Health, College of Medicine, National Cheng-Kung University, Tainan, Taiwan; ^4^Institute of Gerontology, College of Medicine, National Cheng Kung University, Tainan, Taiwan; ^5^Institute of Genomics and Bioinformatics, College of Life Sciences, National Chung Hsing University, Taichung, Taiwan; ^6^College of Education, National Tsing Hua University, Hsinchu, Taiwan; ^7^Research Center for Education and Mind Sciences, National Tsing Hua University, Hsinchu, Taiwan; ^8^Basic Psychology Group, Department of Educational Psychology and Counseling, National Tsing Hua University, Hsinchu, Taiwan; ^9^Department of Kinesiology, National Tsing Hua University, Hsinchu, Taiwan; ^10^Graduate Institute of Mind, Brain, and Consciousness, Taipei Medical University, Taipei, Taiwan

**Keywords:** resilience, dose–response, aging, well-being, adversity

## Abstract

**Background:**

Encountering challenges and stress heightens the vulnerability to mental disorders and diminishes well-being. This study explores the impact of psychological resilience in the context of adverse events, considering age-related variations in its influence on well-being.

**Methods:**

A total of 442 participants (male vs. female =48% vs. 52%) with a mean age of 41.79 ± 16.99 years were collected and completed the following questionnaires Brief Betrayal Trauma Survey (BBTS), Brief Resilience Scale (BRS), Peace of Mind (PoM), The World Health Organization Quality of Life-BREF (WHOQOL-BREF), and Social Support Questionnaire (SSQ). They all underwent structural and resting-state functional magnetic resonance imaging (MRI) scans.

**Results:**

Participants were categorized based on adversity levels: 34.39% faced one, 26.24% none, and 19.91, 9.50, and 8.14% encountered two, three, and four adversities, respectively. This categorization helps assess the impact on participants’ experiences. As adversity factors increased, PoM decreased. Controlling for age improved PoM model fit (Δ*R*^2^ = 0.123, *p* < 0.001). Adversity factors and age explained 14.6% of PoM variance (df = 2, *F* = 37.638, *p* < 0.001). PoM decreased with more adversity and increased with higher age.

**Conclusion:**

The study found most participants faced at least one adversity. Adversity negatively affected PoM scores, while resilience acted as a protective factor. Resilience plays a crucial role in buffering the impact of adversities on well-being. Among those with high adversity, higher resilience correlated with stronger DMN-right frontal pole connectivity. Brain volume showed no significant differences, but the quality of life and social support varied between subgroups, with no differences in personal demographic and biophysical features.

## Introduction

In the contemporary context of our dynamically evolving society, individuals are confronted with many unforeseen adverse events and stressors, which persistently manifest across their lifespans. Amidst the ongoing processes of globalization, modernization, and technological advancements, individuals find themselves in a perpetual state of adaptation, calling for the implementation of effective coping strategies. Daily encounters with adverse events and stressors exert considerable influence on their well-being, exerting effects on mental and physical health, life satisfaction, and overall quality of life (Luhmann et al., 2012). Prolonged exposure to stressors can disrupt individuals’ equilibrium and contribute to symptoms of anxiety, depression, and other psychological disorders (McMahon et al., 2003; Schneiderman et al., 2005). Epidemiological evidence also suggests a link between adverse events and various mental disorders, such as childhood adversities and schizophrenia spectrum disorders or job strain and depressive disorders (Stansfeld et al., 2012; Dragioti et al., 2022).

In practical scenarios, individuals frequently confront the confluence of multiple adverse events and stressors rather than an individual factor. Despite this common occurrence, the interplay between the cumulative impact resulting from multiple adversity factors and its implications on subjective well-being remains uninvestigated. Thus, this study aims to systematically investigate this relationship by quantifying the number of adversity factors and see how the quantity of adversity factors may interact with the relationship. In this study, we adopted the concept of “the dose–response function” from pharmacology and toxicology that describes the relationship between the dose of a substance administered and the biological response it produces (Crump et al., 1976). Specifically, we refer to the dose–response function of adversity factors as the relationship between the ***number*** or ***dose*** of adverse experiences or stressors and their impact on subjective well-being.

This study encompasses an array of adversity factors, which encompass a diverse spectrum of negative life events, traumatic experiences, chronic stressors, and adverse environmental conditions that individuals may encounter. To evaluate these various adversity factors, we utilized the Brief Betrayal Trauma Survey (BBTS), a self-report instrument specifically designed to assess stressful events. The BBTS is designed to capture a wide spectrum of and traumatic events that have occurred during childhood and adulthood. It’s been recognized that the traumatic events that occur during childhood to early adulthood are associated with a heightened risk of chronic health issues, mental health disorders, and substance use problems during both adolescence and adulthood (Felitti et al., 1998). Additionally, early childhood traumatic events have been recognized the exert adverse effects on educational attainment, employment prospects, and overall earning potential. Including those that the participants might not consciously remember. The BBTS’s design allows for evaluating both remembered and potentially unrecognized or repressed adverse experiences. Moreover, this study delved into the presence of chronic diseases by relying on self-reported data from the participants. Chronic diseases under scrutiny included conditions such as heart disease, hypertension, diabetes, leukemia, and other relevant medical conditions. The exploration of chronic diseases provided valuable insight into the potential association between physical health and the impact of adversity on subjective well-being.

The dose–response relationship of adversity factors indicates that as the quantity or intensity of adversities experienced by individuals rises, there is a corresponding escalation in the detrimental effect on their subjective well-being. Consequently, those exposed to a higher number of adversities are more susceptible to manifesting adverse outcomes in comparison to individuals with a lower count of exposures. Furthermore, the form of the dose–response function can differ based on the outcomes under examination and the nature of the adversities in question. The relationship between adversity factors and their impact on subjective well-being may exhibit varying patterns. In certain instances, the association follows a linear trajectory, with the negative impact steadily escalating in proportion to the rising levels of adversity. Conversely, in other cases, the relationship may be nonlinear, signifying that the effects become more pronounced at higher levels of adversity or that a threshold exists beyond which the consequences become notably more severe. These distinctive patterns highlight the complexity of how multiple adversity factors can influence subjective well-being and underscore the need for comprehensive research to explore these nuanced dynamics further.

The shape of the dose–response function can vary depending on the specific outcome being studied, therefore, in this study, we focused on subjective well-being as the outcome evaluation. Previous studies have demonstrated the role of peace of mind (PoM) in predicting well-being (Lee et al., 2013; Chen, 2017). PoM represents a serene and harmonious internal state characterized by low-arousal positive affect and the pursuit of a harmonious state of happiness. In addition, quality of life could be considered as a dimension of subjective well-being. Research has shown that individuals in different cultures prioritize low-arousal positive effects, such as calmness and peace, and these inner states play a crucial role in adapting to global changes and environmental challenges. Studies have linked low-arousal positive affect to decreased levels of depression, anxiety, stress, and increased life satisfaction (Kreitzer et al., 2009; McManus et al., 2019). In this study, we used PoM scores as the dependent variable for the dose–response function to represent the subjective sense of well-being, whereas we used the WHOQOL-BREF scores to identify potential differences in personal characteristics between the high versus the low-resilience subgroups (elaborated in the latter paragraph). The rationale of this design is that PoM reflects a more internal personal feeling which may serve better as a ‘response’ to the dose-effect, whereas WHOQOL-BREF consists of both internal and external indicators of well-being, which may serve better for delineating the differences between low vs. high-resilience individuals.

### Age effect on dose–response function between the number of adversity factors and subjective well-being

Apart from the number of adversity factors, the stage of the life course, represented by age, may also influence the dose–response function of the impact of adversity factors on subjective well-being. Different life stages expose individuals to varying proportions of adverse events. For example, childhood may involve vulnerability to child abuse or neglect, young adulthood may entail more physical accidents, and older adulthood may bring health problems and spousal loss (Smith et al., 2018). Challenges faced throughout life differ significantly, with distinct effects on physical well-being and mental health (Smith et al., 2008). Challenges encountered throughout the various stages of life have significant and distinct effects on both physical well-being and mental health (Smith et al., 2008). As adversity factors accumulate throughout the lifespan, it is likely that their number increases with age. For instance, younger adults are more likely to experience few or no adverse events, while older adults may encounter a higher number of adversity factors. However, population research suggests that the diversity of adverse events tends to increase until middle-aged adulthood and then stabilizes thereafter (Luo et al., 2016). This study thus aimed to explore the issue of whether age modulates, linearly or non-linearly, the relationship between the dose of adversity factors and subjective well-being.

### Resilience effect on dose–response function between the number of adversity factors and subjective well-being

Adverse events have been associated with mental illness, particularly impacting psychological health later in life (Richardson et al., 2023). However, not all individuals experience the same negative outcomes despite facing similar levels of adversity. Resilience plays a crucial role as a protective factor, influencing individual differences in coping with challenging situations. Resilience involves the ability to adapt positively to difficulties. Several resilience scales, such as the Brief Resilience Scale (BRS) (Smith et al., 2008), Resilience Scale for Adults (RSA) (Friborg et al., 2003), and Connor-Davidson Resilience Scale (CD-RISC) (Connor and Davidson, 2003), have been developed to measure psychological resilience. Higher levels of psychological resilience are associated with greater life satisfaction, positive emotions, and subjective well-being (Connor and Davidson, 2003; Windle, 2011). Among these three resilience scales, this study adopted BRS since it emphasizes more on the dynamic process of positive adaption. Resilient individuals experience lower distress, better mental health outcomes, and higher subjective well-being (Smith et al., 2008). Therefore, this study aimed to systematically explore how individual differences in resilience moderate the relationship between adversity factors and well-being. Specifically, the study examined whether individuals with higher levels of resilience exhibit greater positive adaptation, as reflected in subjective well-being, even when facing multiple adverse events.

Yet, while resilience is an important factor in modulating the dose–response function, other individual differences in personal profile, social support, and potential protective factors may also influence this relationship. Therefore, it is worth further investigating potential differences in personal characteristics, including brain (structural and functional) features, between the high and low resilience subgroups among participants who have experienced a high number of adversity factors (i.e., 4–5 adverse events). Understanding the neural underpinnings of resilience can provide valuable insights into the mechanisms by which individuals positively adapt to adversity despite facing significant. According to a study by Eaton et al., gray matter volumes (GMV) in the middle and superior frontal regions have been associated with resilience (Eaton et al., 2022). Additionally, subcortical regions, such as the amygdala and hippocampus, have also been implicated in resilience. Apart from GMV, Eaton et al. (2022) also suggested a potential relationship between brain functional connectivity patterns, particularly involving the Default Mode Network (DMN), and an individual’s resilience. Therefore, in this study, we also investigated if there is a positive relationship between the connectivity of the DMN with other brain regions or networks and the capacity of individuals to exhibit resilience. This suggests that individuals with stronger and more efficient connections between the DMN and other brain regions are likely to demonstrate higher levels of resilience in the face of adversity or challenging situations.

In summary, this study has four main objectives. Firstly, it aimed to examine how the number (analogous to “dose”) of adverse events experienced impacts subjective well-being. Secondly, it sought to explore how age, representing different life course stages, influenced the association between adversity factors and subjective well-being. Thirdly, the study investigated how individual variations in resilience moderated the relationship between the number of adversity factors and well-being. Finally, the study aimed to identify potential differences in personal characteristics, including brain (structural and functional) features, between the high versus the low-resilience subgroups among participants who experienced a high number of adversity factors.

## Materials and methods

### Ethical approval and consent to participate

The study was approved by the Research Ethics Committee (REC) at National Cheng Kung University (NCKU No. 109–419) and the Institute of Review Board (IRB, JA-109-95) of Jen-Ai Hospital. All participants were given a full explanation of the study and signed an informed consent form agreeing to join the research.

### Participants

We contacted a total of 547 participants in southern Taiwan through various channels, including internet advertisements, bulletin board notices, and distributing flyers around the university. However, out of the initial participant pool, 105 individuals chose not to participate in the experiment after receiving detailed explanations of the methodology and conditions during phone consultations or due to other reasons. These reasons included time constraints, left-handedness, previous incidents of brain injuries, or existing mental illnesses. Participants’ medical information, encompassing neurological history and mental health status, was collected through self-reports. Consequently, the final sample size for analysis comprised 442 right-handed individuals, determined by the Edinburgh Handedness Inventory (Oldfield, 1971), with no prior history of psychiatric or neurological disorders. Therefore, they were considered healthy participants.

The age range of the participants was 20 to 80 years old, with a mean age of 41.79 ± 16.99 years (standard deviation, SD). The gender distribution among the participants was 52% female. Before their involvement in the study, all participants were provided with a written informed consent form approved by the REC of the university and the IRB of the hospital. They willingly signed the consent form to confirm their agreement to participate in the study.

During the study, all participants were required to complete the questionnaires collect personal demographic information and assess their resilience score and well-being measurements. Following the completion of the questionnaires, the participants underwent brain imaging scans using a 3 T MRI scanner. Prior to undergoing the MRI procedure, participants were subjected to a thorough screening process. This process involved assessing factors such as the presence of metal implants, pacemakers, or other contraindications that could compromise the safety of the individual during the scan. Additionally, participants were required to provide information regarding any history of claustrophobia or anxiety, as this could impact their ability to undergo the MRI comfortably. The screening criteria aimed to identify any potential risks or conditions that might contravene the safety protocols of the MRI scan, allowing for a comprehensive evaluation of participants’ eligibility and the overall success of the imaging procedure. Despite these precautions, 35 of the 422 participants were excluded from the MRI analysis. Exclusions were due to various factors, including body or eyebrow tattoos, discomfort in the MRI machine, and scan cancelations. This meticulous screening process was crucial for the integrity and success of our study’s imaging component.

After completing the MRI scans and questionnaires, each participant received compensation as a token of appreciation for their time and contribution to the study. The amount of compensation varied based on the extent of their participation. Participants who completed the MRI scans and questionnaires received a fixed compensation of US$80. However, for those who only completed the questionnaires, their compensation was adjusted proportionally based on the time they spent participating in the study. This approach ensured that all participants were appropriately recognized for their valuable contributions.

### Personal information collection

During the study, personal information was collected to gather basic demographic data and assess relevant factors. The information collected included the participants’ age, gender, height, weight, waist circumference, and blood pressure (systolic and diastolic measurements). Height and weight were utilized to calculate the Body Mass Index (BMI), further analyzed as part of the study.

In addition to the physical measurements, information regarding the participants’ income level and monthly expenditure was also collected. This data aimed to provide insights into the participants’ financial situations and potential economic influences on the study’s outcomes. Furthermore, the survey included questions about the participants’ exercise habits, such as frequency and intensity, as well as whether they had any chronic diseases or pre-existing medical conditions. Collecting this personal information enabled a comprehensive assessment of various factors that could impact the study’s objectives and findings. Strict privacy protocols were followed to ensure the confidentiality and anonymity of the participants’ data.

### Questionnaires

#### The brief betrayal trauma survey (BBTS)

The BBTS is a specific questionnaire developed to distinguish between different types of trauma, with a focus on interpersonal trauma. Its main purpose is to assess and identify the level of closeness in relationships between victims and perpetrators (Goldberg and Freyd, 2006). This specialized survey helps researchers and professionals gain insights into the complexities of traumatic experiences within interpersonal contexts and the dynamics of betrayal in such situations. The BBTS questionnaire consists of a total of 24 items, with 12 items pertaining to experiences before the age of 18 and 12 items relating to experiences after the age of 18. Each item is assessed using a 3-point Likert scale (value of ‘0’ = No occurrence, ‘1’ = 1–2 times, and ‘2’ = above 2 times), with a rating scale of 0 to 7. A higher score on each item indicates a higher frequency of the experienced event. The Mandarin Chinese version of the BBTS was translated by Chiu et al. (2010) to facilitate its use among Mandarin-speaking populations.

#### Peace of mind (PoM)

The Peace of Mind (PoM) questionnaire comprises seven items, with participants rating each on a 5-point Likert scale. This questionnaire is designed to evaluate the level of tranquility experienced in individuals’ minds, characterized by a state of inner calmness and harmony (Lee et al., 2013).

### The World Health Organization Quality of Life-BREF (WHOQOL-BREF)

The WHOQOL-BREF scale, developed by the World Health Organization (WHO), is a tool used to assess an individual’s subjective evaluation of their life satisfaction and overall well-being. The WHOQOL-BREF is a 26-item instrument assessing physical, psychological, social, and environmental domains. It uses a five-point scale to rate items, transformed to a 0–100 scale. Physical health covers mobility, activities, capacity, energy, pain, and sleep. Psychological measures include self-image, thoughts, attitudes, self-esteem, mental well-being, learning, memory, religion, and mental status. Social relationships focus on personal connections, support, and sexual life. Environmental health covers finances, safety, access to services, living environment, learning opportunities, recreation, environment quality, and transportation. This concise tool provides insights into well-being, quality of life, and general health. The Taiwan version of this scale, developed by Yao et al. (2002), aims to measure the individual’s sense of fulfillment and happiness across various aspects of life. These aspects include physical health, psychological well-being, social relationships, and environmental factors.

The questionnaire consists of different domains. The physiological domain, which encompasses physical health and independence-related aspects, includes a total of 7 items. The psychological domain, covering mental, spiritual, religious, and personal belief aspects, consists of 6 items. The social relationship domain comprises 4 items, while the environmental domain includes 9 items. Participants were asked to rate their level of satisfaction on a scale ranging from 1 to 5, where 1 represents “very dissatisfied” and 5 represents “very satisfied.”

To calculate the score for a specific domain, the following formula is used:

$\left(\begin{array}{l} \mathrm{Sum}\;\mathrm{of scores for}\;\mathrm{all}\\ \mathrm{items in the domain} \end{array}\right)\times \;4/\left(\begin{array}{l} \mathrm{Number of items}\\ \mathrm{in the domain} \end{array}\right)$

### The social support questionnaire (SSQ)

The Social Support Questionnaire (SSQ) was originally developed with 27 items to assess an individual’s perception of social support and their satisfaction with the received support (Sarason et al., 1983). For the Mandarin version, Wu (1985) translated the questionnaire, which was subsequently shortened and modified to 20 items by Chang (1989). This questionnaire measures two primary dimensions: (a) the quantity of social support and (b) individuals’ satisfaction with the social support they receive. The internal reliability of the Mandarin version of SSQ [SSQ (N)] was with a Cronbach’s alpha of 0.91, indicating good consistency among the items measuring social support. Similarly, for the satisfaction subscale [SSQ (S)], the internal reliability was also high, with a Cronbach’s alpha of 0.92. The SSQ is a valuable instrument utilized to evaluate social support among individuals.

### The brief resilience scale (BRS)

The BRS developed by Smith et al. in 2008 was utilized in this study. The BRS was developed to evaluate the perceived capacity to rebound or recover from stress and was designed to assess a unitary construct of resilience. The scale was administered to a sample of 354 individuals and consisted of six items (e.g., I tend to bounce back quickly after hard times), each rated on a five-point scale. Three items are positively worded, while the remaining three are negatively worded. Resilience, as defined by the BRS, refers to the ability to rebound from stressful experiences effectively. To ensure accuracy, reverse scoring was performed for the negatively worded items, and the average score was calculated for each participant. A higher score on the BRS indicates a greater level of resilience in coping with stress. The Chinese version of the BRS was translated and subjected to validity testing by Hsin and their team in 2020.

### Adversity factor: quantifying both the number and the type

The BBTS was utilized to assess self-reported stressful events and evaluate four specific types of adversity factors.

The first factor, “Experiencing Disasters,” was recorded as 1 if the participant reported having experienced natural disasters or traffic accidents, otherwise 0.

The second factor, “Traumatic life experiences,” was recorded as 1 if the participant reported intentional harm, forced sexual contact, emotional oppression, or psychological abuse after the age of 18, otherwise 0.

The third factor, “Being abused or neglected as a child,” was recorded as 1 if the participant experienced intentional harm, forced sexual contact, emotional oppression, or psychological abuse before the age of 18, otherwise 0.

The fourth factor, “Loss, either by death, divorce, or other means,” was recorded as 1 if the participant reported witnessing or experiencing severe harm, suicide, homicide, divorce, or separation of a close or non-close person, otherwise, 0.

Additionally, for the fifth factor, “Biophysical,” each participant was surveyed about the presence of chronic diseases such as heart disease, hypertension, diabetes, leukemia, etc. If they reported having any chronic disease, it was recorded as 1, otherwise 0.

The total score for these five factors was calculated as the number of adversity factors reported by each participant. In addition, we also calculated the proportion of participants who reported experiencing each adversity factor by dividing the number of participants who indicated the presence of that specific adversity by the total number of participants in the study.

### Structural and functional image acquisition

Magnetic resonance imaging (MRI) structural images were acquired using a GE MR750 3 T scanner (GE Healthcare, Waukesha, WI) located at the Mind Research Imaging Center at National Cheng Kung University. High-resolution structural images were obtained using a fast-SPGR sequence, comprising 166 axial slices [TR/TE/flip angle: 7.6 ms/3.3 ms/12°; field of view (FOV): 22.4 × 22.4 cm^2^; matrix size: 224 × 224; slice thickness: 1 mm]. The entire scanning procedure lasted approximately 218 s.

Resting-state functional imaging data were collected using an interleaved T2*-weighted gradient-echo planar imaging pulse sequence. The imaging parameters used were as follows: a repetition time (TR) of 2000 ms, an echo time (TE) of 30 ms, and a flip angle of 77°. The matrices size was 64 × 64, the field of view (FOV) was 22 × 22 cm^2^, the slice thickness was 4 mm, and the voxel size was 3.4375 × 3.4375 × 4 mm. A total of 245 volumes were acquired to cover the entire brain of each participant. To establish steady-state magnetization, the scanner performed 5 dummy scans, which were discarded and not included in the subsequent analysis.

During the resting-state functional scans, participants were instructed to remain awake with open eyes, focusing on a white cross displayed on a screen. The total scanning time per participant was 8 min and 10 s, calculated as [(number of samples + number of dummy scans) × TR = (240 + 5) × 2 = 490 s].

### Structural MRI image preprocessing

To estimate regional gray matter (GM) volumes, we utilized FreeSurfer 5.3^1^. For the 3 T MRI scans, we employed the recon-all flag in FreeSurfer, which includes an N3 bias field correction parameter suitable for our research purposes. Neuroanatomical labels from the Desikan-Killiany Atlas^2^ were utilized to define regions of interest (ROIs) and map them onto a cortical surface model. GM volumes within each ROI were extracted from the output aseg.stats and aparc.stats files provided by FreeSurfer’s atlas. Based on previous research (Eaton et al., 2022), which has identified associations between resilience and specific brain regions such as the middle frontal cortex, superior frontal cortex, amygdala, and hippocampus, we selected these regions as our ROIs of interest for this study.

### Functional MRI image preprocessing

For the preprocessing of the functional images, we employed the CONN toolbox 18a^3^ and SPM 12^4^ within Matlab (The MathWorks, Inc., Natick, MA, United States). Our preprocessing protocol was adapted from Geerligs and Tsvetanov's (2017) study (see also Hsieh et al., 2021).

The initial preprocessing step involved several procedures: slice timing correction, realignment, normalization (using a T1 image for registration to standard space), and smoothing with an 8-mm Gaussian kernel. Additionally, the images were resliced to a voxel size of 2 × 2 × 2 mm, resulting in a data cube with dimensions of 91 × 109 × 91 voxels.

In the second step, we calculated nuisance covariates (R) that included various noise sources. These covariates consisted of movement parameters (translations along the x, y, and z axes, as well as rotations along roll, yaw, and pitch directions), white matter (WM) signals, and cerebral spinal fluid (CSF) signals.

The third step involved regressing out bad frames at the subject level. These frames were identified through “head motion censoring” and [R R2 Rt-1 R2t-1], where t and t-1 represent the current and immediately preceding time points, respectively.

Finally, a band-pass filter was applied simultaneously to the nuisance covariates and fMRI data in the last preprocessing step. The band-pass filter ranged from 0.008 to 0.1 Hz, allowing noise removal while retaining the relevant frequency range of interest.

### Statistical analyses

All analyses were conducted with the JASP package [JASP Team (2021). JASP (Version 0.15) (Computer software)].

### The impact of the number of adversity factors on PoM scores

To examine the impact of the number of adversity factors on Peace of Mind (PoM) scores, a stepwise multiple regression analysis was conducted. The purpose was to investigate whether the relationship between PoM scores and the number of adversities exhibits a linear or nonlinear pattern. The regression model included two predictors: the number of adversity factors and the squared number of adversity factors (number of adversity factors^2^). These predictors were added to the regression model sequentially, and the change in R^2^ (ΔR^2^) was examined for significance. This analysis enabled us to explore potential nonlinear associations between the number of adversities and PoM scores. The formula is shown below:

$$\begin{array}{l} \mathrm{Well-being}=\mathrm{number of adversity factors}\\ \;+\mathrm{number of}\;{\mathrm{adversity factors}}^{2} \end{array}$$

### Dose–response effects of the number of adversity factors on PoM scores: step-wise multiple regression

To explore the potential influence of age and resilience on the dose–response effects of adversity factors on Peace of Mind (PoM) scores, two sets of step-wise multiple regression models were conducted. These models were designed to examine how age and resilience may independently contribute to the relationship between the number of adversity factors and individuals’ levels of Peace of Mind.

The first set of models explored the relationship between the number of adversity factors and age on well-being. The regression equation was defined as follows:

$$\mathrm{Well-being}=\mathrm{number of adversity factors}+\mathrm{age}+{\mathrm{age}}^{2}$$

Age was treated as a continuous variable and included linear and polynomial terms. The predictors, including the number of adversity factors, age, and age^2^, were sequentially added to the regression model. The change in R^2^ (ΔR^2^) was then assessed for significance, providing insights into the dose–response relationship between the number of adversity factors and PoM scores based on different age levels.

The second analysis aimed to examine the dose–response relationship between the number of adversity factors and resilience (measured by the BRS score) on well-being. The regression equation for this analysis was defined as follows:

$$\begin{array}{l} \mathrm{Well-being}=\mathrm{age}+\mathrm{number of adversity factors}\\ \;+\mathrm{resilience}+{\mathrm{resilience}}^{2} \end{array}$$

Initially, age was entered into the model as a covariate. Subsequently, the number of adversity factors, resilience, and resilience^2^ were included in the model. The change in R^2^ (ΔR^2^) was then examined for significance, providing insights into the dose–response effect of the number of adversity factors on PoM scores while considering the influence of age and resilience.

### Contrasting the personal profile between high vs. low resilient subgroups among participants who experienced a high number of adversity factors: Bayesian *t*-test

To gain deeper insights into the factors contributing to the capacity of individuals with high resilience to maintain well-being despite facing numerous adversity factors, a Bayesian *t*-test was conducted. This analysis aimed to explore the differences in individual characteristics between the high resilience subgroup and the low resilience subgroup within participants who encountered a high number of adversity factors, specifically ranging from 4 to 5 factors. By using a Bayesian approach, the study aimed to better understand the distinct attributes that might be associated with high resilience in the face of significant adversity.

To refine the analysis and ensure comparability, specific criteria were applied for participant selection. In the low resilience subgroup, only participants with well-being scores having a standard deviation below 0 were included. Similarly, in the high resilience subgroup, only participants with well-being scores having a standard deviation greater than 0 were included.

To control for the influence of age (while excluding gender), a regression analysis was performed to remove the effect of age from the data. Following the age regression, the standardized residuals were utilized for comparing the two groups.

By employing Bayesian t-tests, we sought to elucidate the differences in personal profiles (e.g., gender, education, BMI, blood pressure, waistline, monthly cost, income, sport, WHOQOL-BREF, SSQ) between the high and low resilience subgroups, providing insights into the underlying factors contributing to the resilience of individuals who maintain well-being despite facing multiple adversity factors.

### Contrasting the brain GMV between high vs. low resilient subgroups among participants who experienced a high number of adversity factors: multivariate analysis of variance (MANOVA)

An analysis was conducted on the T1 brain images to compare the high resilience subgroup with the low resilience subgroup among participants who experienced significant adversity factors (e.g., 4–5 adversity factors). Gray matter volumes were extracted from various brain regions to conduct a MANOVA test. However, it is worth noting that in the high resilience group, three individuals only completed the questionnaire assessment and did not undergo brain imaging scans. As a result, the sample sizes for the two groups were 15 and 10 individuals, respectively.

Drawing on the findings of Eaton et al.'s (2022) study, it is established that gray matter volumes in the middle and superior frontal regions are associated with resilience. Subcortical regions such as the amygdala and hippocampus also play a role. To assess the differences between the two subgroups, gray matter volumes in each region were calculated for the participants while controlling for age and total intracranial volume (TIV). Subsequently, a MANOVA analysis was conducted using the residuals to investigate potential group differences.

### Contrasting the brain resting-state fMRI between high vs. low resilient subgroups among participants who experienced a high number of adversity factors: functional connectivity analysis

Seed-based connectivity maps (SBC) were estimated using 32 High-Performance Computing Independent Component Analysis (HPC-ICA) network ROIs to characterize functional connectivity patterns. The strength of functional connectivity was quantified using Fisher-transformed bivariate correlation coefficients derived from a weighted General Linear Model (weighted GLM). This model was applied individually for each pair of seed and target areas to model the relationship between their respective BOLD signal time series.

Based on the study by Eaton et al. (2022), which suggests that the connectivity between the DMN and other brain regions or networks may play a crucial role in resilience, the first-level connectivity measures using the DMN as the seed was selected for further investigation.

Group-level analyses were conducted using the Generalized Linear Model (GLM). For each individual voxel, a separate GLM was estimated with the first-level connectivity measures of that voxel as the dependent variable, group as the independent variable, and age as a covariate. The between-subjects contrast vector [1, −1, 0] was used for two groups and age. Voxel-level hypotheses were evaluated using multivariate parametric statistics, considering random effects across individuals and sample covariance estimation across multiple measurements. Inference was performed at the level of individual clusters, which are contiguous groups of voxels. The cluster-level inference was based on parameter statistics using Gaussian Random Field theory. The results were thresholded using a voxel-level threshold of *p* < 0.001 to form clusters, and a cluster size threshold of *p* < 0.05 after False Discovery Rate (FDR) correction.

## Results

### The number and proportion of participants for different numbers of adversity factors

**Figure 1:**
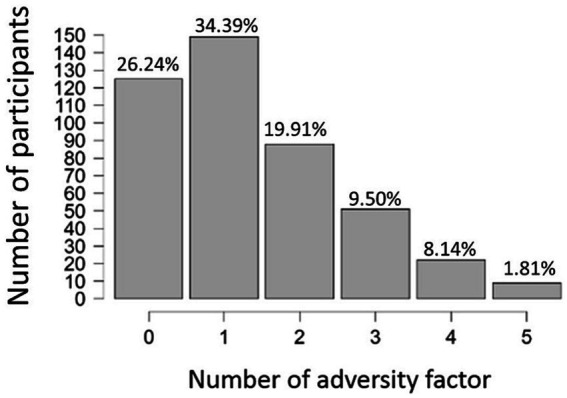
The number (the *x*-axis) and proportion (denoted on the top of each bar) of participants who had experienced how many numbers (0 ~ 5) of adverse events (the *x*-axis).

Among the participants, the largest proportion, comprising 34.39% (*n* = 152), experienced one type of adversity. The second-largest group, comprising 26.24% (*n* = 116), had not encountered any adversity. Individuals facing two types of adversities accounted for 19.91% (*n* = 88), while those experiencing three types represented 9.50% (*n* = 42) of the total sample. Participants who encountered four types of adversities made up 8.14% of the total sample (*n* = 36). The smallest proportion was observed among individuals who experienced five types of adversities, accounting for only 1.81% (*n* = 8) of the sample (see Figure 1).

**Figure 2:**
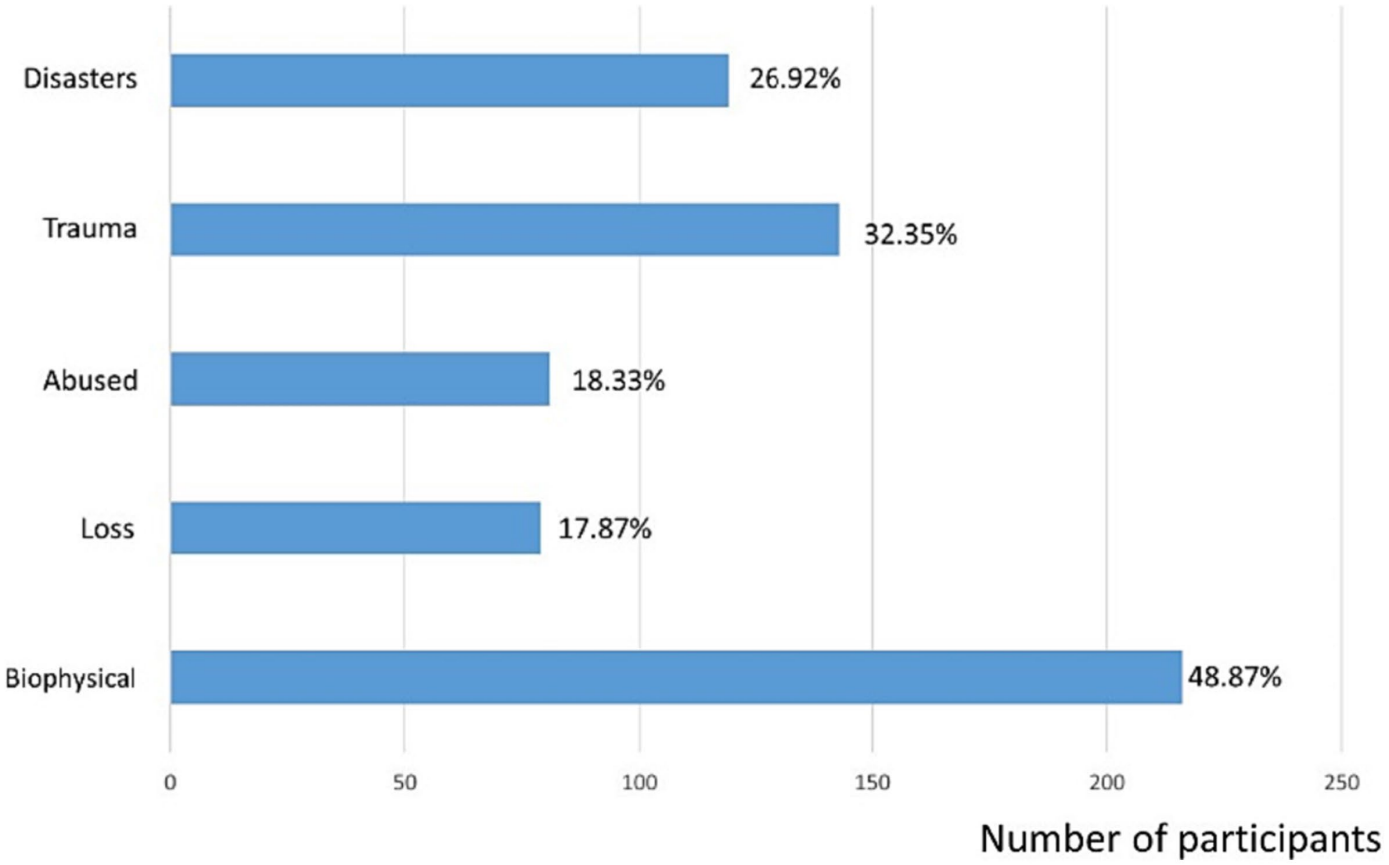
The number (the upper *x*-axis) and proportion (the lower *x*-axis) of participants who indicated the presence of that specific adversity (the *y*-axis).

Among all participants, the most commonly experienced adversity factor is “Biophysical” with 48.87% of participants (*n* = 216) reporting related adversities. This factor is followed by “Traumatic life experiences” (*n* = 143, 32.35%) and “Experiencing Disasters” (*n* = 119, 26.92%). Factors such as “Being abused or neglected as a child” (*n* = 81, 18.33%) and “loss, either by death, divorce, or other means” (*n* = 79, 17.87%) were less frequently experienced by participants (see Figure 2).

### The impact of the number of adversity factors on PoM scores

Stepwise hierarchical regression analysis reveals that adding the number of adversity factors to the model significantly improves the model fit compared to a model that includes only intercept (model 1: Δ*R*^2^ = 0.023, *p* = 0.001). But incorporating the quadratic term for the number of adversity factors did not significantly increase the explained variance in the model (model 2, Δ*R*^2^ = 0.006, *p* = 0.102).

**Figure 3:**
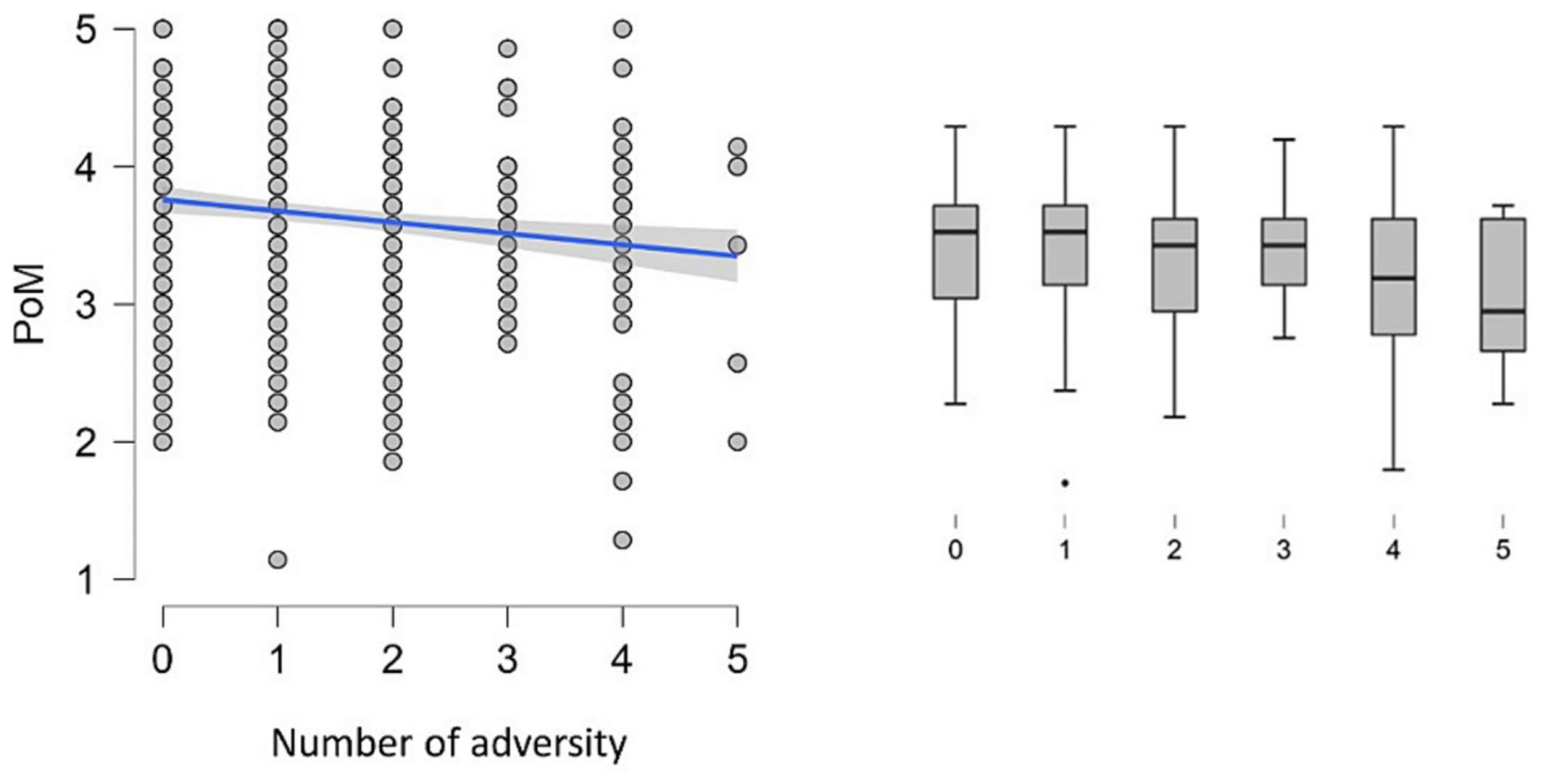
The left panel shows the scatter plot of Peace of Mind (PoM) and the number of adversities. The blue line represents the negative correlation. The right panel shows the boxplot for each number of adversity factors (*x*-axis) against PoM scores (*y*-axis). The box represents the data’s interquartile range (IQR), with the central line within the box indicating the median PoM score. The “whiskers” extend to the highest and lowest values within 1.5 times the IQR. Data points beyond the whiskers are considered outliers and plotted individually as dots.

The initial analysis, with the PoM score as an outcome measure, revealed a significant impact of the number of adversity factors on the PoM score. As the number of adversity factors increases, PoM decreases. No nonlinear relationship exists between PoM and the number of adversities (see Figure 3).

### Dose–response relationship of the number of adversity factors and age on PoM scores

The stepwise hierarchical regression analysis demonstrates that including age in the model significantly enhances model fit in comparison to a model containing only the number of adversity factors (model 1: ΔR^2^ = 0.023, *p* = 0.001). Furthermore, the inclusion of age (model 2, Δ*R*^2^ = 0.123, *p* < 0.001) enhanced the model’s ability to increase the percentage of variances explained. However, including the quadratic term for age did not significantly increase the explained variance in the model (model 3, Δ*R*^2^ = 0.000, *p* = 0.901). The result hence suggests Model 2 was the best-fitting model.

**Figure 4:**
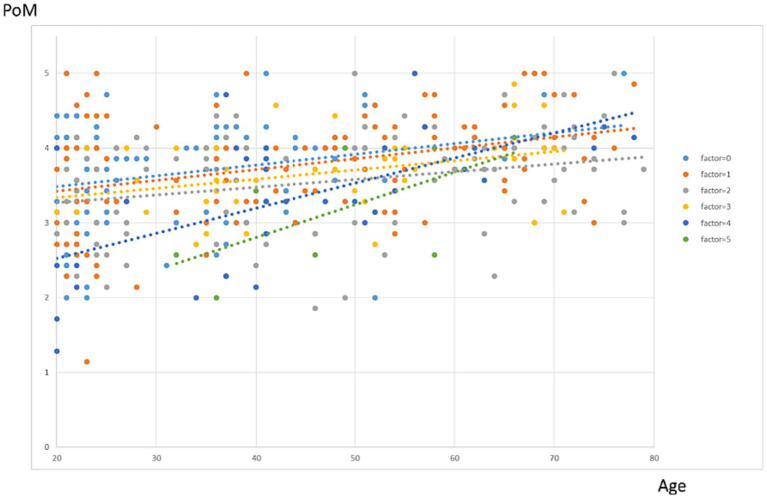
Dose–response effects of the number of adversity factors on PoM score per age. Each color represents a different number of adversity factors. The lines represent fitted polynomial curves.

In model 2, the number of adversity factors, and age explained 14.6% of the variance in PoM Scale (df = 2, *F* = 37.638, *p* < 0.001). PoM Scale exhibited a significant decrease as the number of adversity factors increased (*β* = −0.126, *p* < 0.001), while it increased with higher age (*β* = 0.015, *p* < 0.001). The results indicate that age influences the dose–response function of the number of adversities on PoM scores (see Figure 4).

### Dose–response relationship of the number of adversity factors and BRS scores on PoM scores

The initial analysis revealed a significant impact of the number of adversity factors and resilience scores on the PoM scores. As the number of adversity factors increases, PoM scores decrease, while resilience leads to an increase in PoM scores, counteracting the effects of adversity factors.

When controlling for age, a stepwise hierarchical regression analysis demonstrated that adding BRS scores significantly improves the model’s fit and enhances its ability to explain more variance compared to a model that includes age and the number of adversity factors (model 1: Δ*R*^2^ = 0.146, *p* < 0.001).

Furthermore, the inclusion of BRS (model 2, Δ*R*^2^ = 0.224, *p* < 0.001) enhanced the model’s ability to explain more variance. However, including the quadratic term for BRS did not significantly increase the explained variance in the model (model 3, Δ*R*^2^ = 0.000, *p* = 0.627). The result hence suggests Model 2 was the best-fitting model.

In model 2, age, number of adversity factors and BRS explained 37.0% of the variance in PoM scores (df = 3, *F* = 85.551 *p* < 0.001), PoM scores exhibited a significant decrease as the number of adversity factors increased (*β* = −0.093, *p <* 0.001). In contrast, it increased with higher BRS scores (*β* = 0.518, *p <* 0.001).

**Figure 5:**
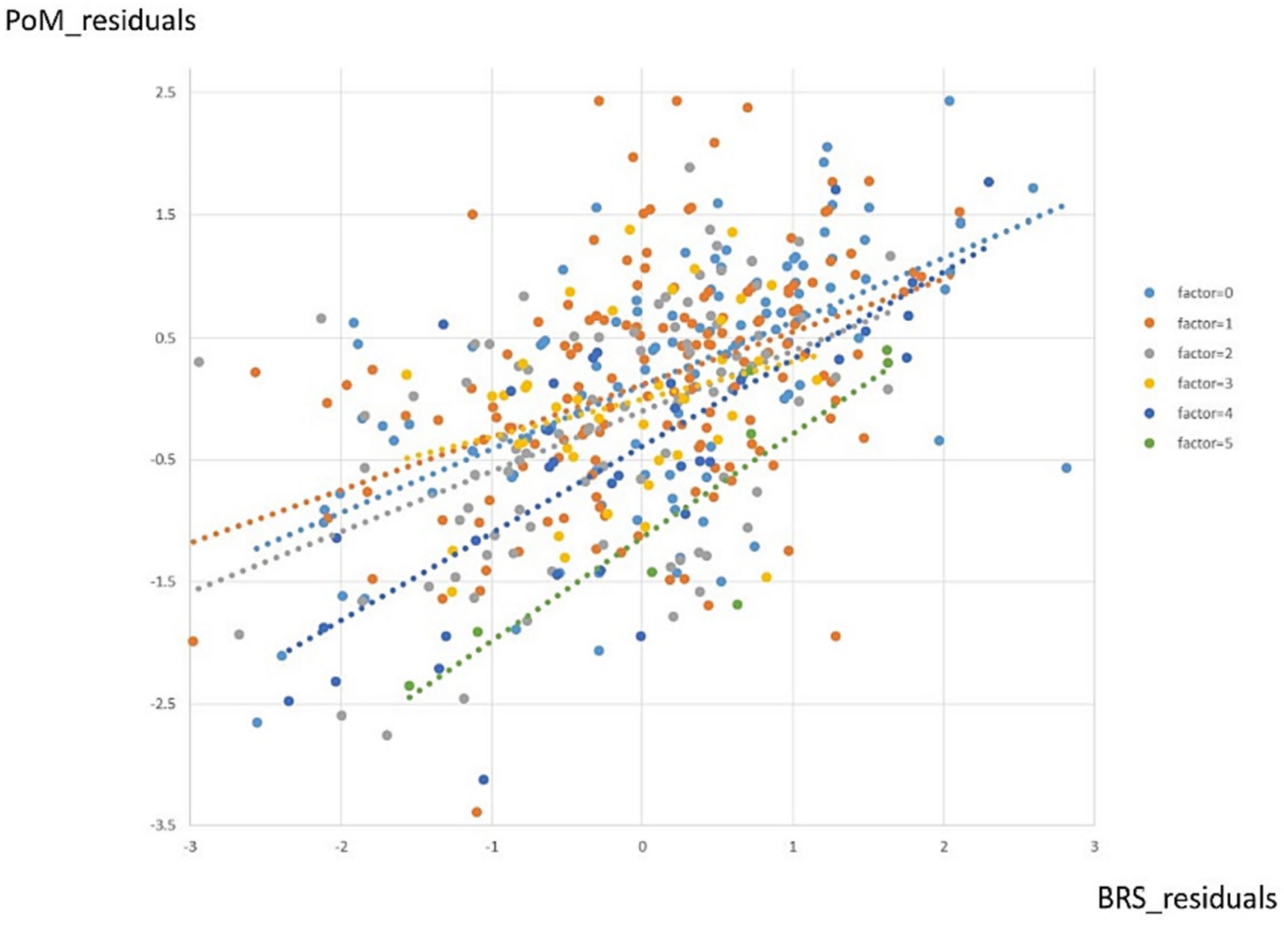
Dose–response effects of the number of adversity factors on PoM score per BRS score. Each color represents a different number of adversity factors. The lines represent fitted polynomial curves.

The findings demonstrated that even after accounting for age, resilience plays a significant role in buffering the impact of adversity factors on well-being. Highly resilient individuals can maintain a similar sense of well-being despite facing a greater number of adversity factors, in contrast to less resilient individuals who experience fewer or no adversity factors. This indicates that resilience acts as a protective factor, enabling individuals to resist the negative effects of adversities on their well-being (Figure 5).

### Contrasting the personal profile between high vs. low resilient subgroups among participants who experienced a high number of adversity factors

A total of 39 participants who have experienced a high number of adversity factors (i.e., the number of adversity factors = 4, 5) were divided into two subgroups based on a medium split, representing high and low resilience. Among the 17 individuals in the low resilience subgroup (BRS = 1.66 ~ 3), 2 individuals had a standard deviation of PoM > 0. Therefore, only 15 individuals were included in the subsequent analysis. Similarly, among the 22 individuals in the high resilience subgroup (BRS = 3.16 ~ 5), 9 individuals had a standard deviation of PoM < 0. Therefore, only 13 individuals were included in the subsequent analysis (see Table 1).

**Table 1:** Bayesian *T*-test results for two subgroups within a high number of adversity factors experienced by participants.

	BF10	Error%
Individual’s personal life information		
gender	0.361	1.97*10–5
education	1.068	0.002
BMI	0.397	2.29*10–4
SBP	0.491	7.89*10–5
DBP	0.376	8.52*10–5
waistline	0.586	0.001
Monthly cost	0.395	2.16*10–4
income	2.655	0.001
Sport frequency	0.420	3.58*10–4
Questionnaire		
Physical- WHOQOL-BREF	1.790	0.001
Psychological- WHOQOL-BREF	39.920	7.20*10–5
Social_relationship- WHOQOL-BREF	1.554	7.56*10–4
Environment- WHOQOL-BREF	3.033	7.64*10–4
SSQ	0.813	0.005

Ps. The error % is based on the accuracy of the Bayes factor calculations, if this is less than 10% this can be ignored. SBP (Systolic blood pressure), DBP (Diastolic blood pressure), SSQ (Social support Questionnaire). The Bayes Factor (BF10) is a measure of the strength of evidence in favor of the alternative hypothesis compared to the null hypothesis.

Regarding individual personal life information, no significant differences were observed between the two subgroups. The Bayes Factor (BF_10_) for all comparisons was less than 3, with the closest being 2.655 for the variable “payment.” This result indicates that there is 2.655 times more support for the alternative hypothesis (H_1_) compared to the null hypothesis (Group1 = Group2), but it falls short of providing moderate evidence (BF_10_ > 3).

In terms of quality of life and social support aspects, significant differences were observed between the two groups in terms of environment and psychological subscales of the WHOQOL-BREF (BF_10_ > 3), indicating moderate evidence in favor of the alternative hypothesis. However, in other sub-scores (physical, social relationship, SSQ), no significant differences were found (BF_10_ < 3), suggesting that there is insufficient evidence to support a meaningful distinction in personal demographic and biophysical features between the two groups in these aspects. Nevertheless, we further investigate if the brain features might exhibit differences between the two subgroups.

### Contrasting brain features between high vs. low resilient subgroups among participants who experienced a high number of adversity factors

#### Brain structural GMV results

MANOVA analysis was conducted to examine the comparison between the two subgroups of their GMV in the middle frontal and superior frontal regions in both hemispheres of the brain. However, the analysis did not reveal any significant differences in GMV between the two subgroups (see Supplementary Figure S1). MANOVA analysis also examined the differences in subcortical regions between the two groups, but similarly, no significant differences were found (see Supplementary Figure S2).

#### Brain resting-state functional connectivity results

**Figure 6:**
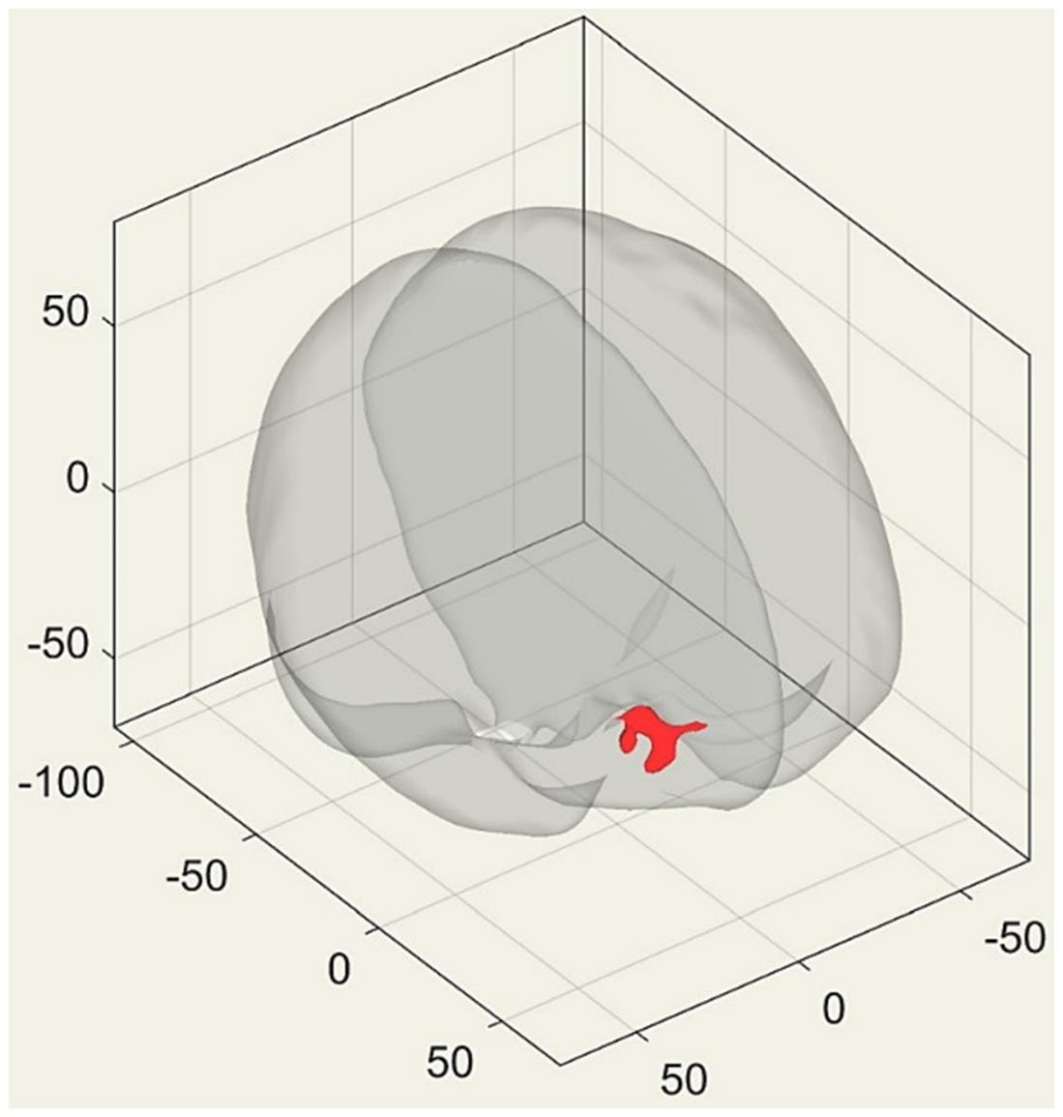
3D transparent brain perspective view, with the red area representing the brain cluster displaying significant differences after the Generalized Linear Model (GLM) contrast. The figure presents the coordinates of brain images in MNI (Montreal Neurological Institute) space.

The analysis revealed a significant difference in functional connectivity between the two sub-groups. Specifically, we observed increased functional connectivity between the DMN and the right frontal pole. The cluster is located at coordinates (24, 46, 16) and has a size of 261 voxels. At the cluster level, the value of p after FDR correction is 0.0095. This indicates that under high adversity, the high-resilience group exhibits stronger functional connectivity between the DMN and the right frontal pole compared to the low-resilience group (see Figure 6).

## Discussion

### Summary of the results

This study aimed to explore the impact of adversity factors on individual subjective well-being, using PoM scores for well-being, BBTS scores for adversity factors, and BRS scores for resilience. Findings revealed a negative correlation, with increasing adversity factors leading to decreased PoM scores, indicating adverse effects on well-being.

The second aim was to examine how age moderates the link between adversity factors and subjective well-being. Results revealed a significant positive impact of age on this relationship. Older adults demonstrated higher subjective well-being regardless of adversity, while younger adults’ well-being depended on the number of adverse events experienced.

The third aim of this study was to explore how resilience impacts the link between adversity factors and well-being. Findings showed that highly resilient individuals maintained similar well-being even when facing multiple adversity factors (e.g., 4–5 adverse events), while less resilient individuals with fewer or no adverse events experienced lower well-being.

The final aim was to explore differences in personal characteristics, including brain (structural and functional) features, between high and low resilience subgroups among participants experiencing a high number of adversity factors (i.e., 4–5 adverse events). The results revealed significant distinctions only in the environment and psychological subscales of the WHOQOL-BREF, while no significant differences were observed in other personal profile indicators (gender, education, BMI, physical, socio-economic status, and social support). Regarding brain features, there were no significant differences in structural GMV between the subgroups, but a significant difference in the resting-state DMN.

### Age effect on dose–response function between the number of adversity factors and subjective well-being

Among the significant findings of this study, the most intriguing one is the demonstration, for the first time, of a dose–response association where each additional adversity factor was linked to lower subjective well-being but with variations across age. This suggests that older adults, compared to younger adults, exhibited a positive bias in subjective well-being despite experiencing numerous adverse events. These results align with previous literature indicating a prominent positivity effect, wherein older individuals tend to favor positive information over negative information during old age (Ziaei et al., 2015).

Various studies investigating different cognitive aging functions have presented compelling evidence supporting the positive emotion bias observed in older adults (Mroczek and Kolarz, 1998). For instance, research consistently indicates that older adults prefer attending to and remembering positive information over negative information, showing better memory recall and allocating more attention to positive stimuli like positive images or words (Mather, 2012; Nashiro et al., 2012; Ziaei et al., 2015). Older adults also display enhanced emotion regulation abilities, particularly in managing negative emotions, leading to an overall more positive emotional state (Isaacowitz, 2022). Despite facing challenges associated with aging, older individuals tend to maintain higher levels of emotional well-being and life satisfaction, reflecting a positive outlook on life and increased happiness and contentment (Cho and Cheon, 2023). Overall, the cumulative evidence consistently points to a positive emotion bias in older adults, highlighting their inclination toward positive information, enhanced emotion regulation, and higher emotional well-being.

The observed variation in the dose–response effect with age in the current results may be attributed to a sample selection bias. The older adults included in this study needed to possess certain capabilities for task performance and imaging scanning, potentially leading to the selection of individuals associated with successful aging. Prior research, such as that by Paúl (2019), has suggested that successful aging involves an interplay between cognition and emotion, leading to a shift toward positivity and subjectivity in logical thinking throughout the lifespan. This transformation is mainly driven by the learning process. In the case of older adults, there is a distinct tendency to prioritize affective aspects over logical ones. It is essential to note that this preference arises not due to an inability to behave differently, but rather as the result of the adaptive evolution of affect and cognition through learning. Older individuals are inclined to adopt a more affective and subjective approach when engaging with the physical and social environment, contributing to their overall successful aging (Paúl, 2019).

Further supportive studies, like those conducted by Petro et al. (2021), corroborate the notion that older adults tend to exert less effort and respond more quickly when making positive categorizations. Additionally, older adults with a stronger positive bias also show increased amygdala habituation, indicating a reduced perception of potential threats that would require further learning. These findings underscore the pronounced emphasis on positivity during the aging process. It is crucial to recognize that the positivity effect does not solely arise from an automatic or bottom-up process, as other cognitive mechanisms may also be involved.

### Resilience effect on dose–response function between the number of adversity factors and subjective well-being

The second most noteworthy finding of this study is the demonstration, for the first time, of a dose–response association between adversity factors and subjective well-being, which varied according to resilience scores. Highly resilient individuals could maintain a similar sense of well-being despite facing more adversity factors, unlike less resilient individuals who were impacted by even a few or no adverse events. Furthermore, despite encountering more adversity factors, highly resilient individuals can still maintain a similar sense of well-being. On the other hand, low-resilient individuals experienced noticeable differences in their level of well-being when facing varying levels of adversity. This phenomenon can provide complementary evidence of resilience and subjective reports based on resilient scales.

### Contrasting the personal profile and brain features between high vs. low resilient subgroups among participants who experienced a high number of adversity factors

Based on this interesting finding, we further investigated the potential demographic, physical, psychosocial, and brain features contributing to the distinctions between high and low-resilient subgroups among participants who encountered a high number of adversity factors. The current results indicated no significant differences between the two subgroups in certain personal profile indicators, including gender, education, BMI, physical health, socio-economic status, and social support. Additionally, there were no significant differences in brain GMVs between the subgroups. However, a significant difference was observed in the connectivity of the DMN between the two subgroups, suggesting that the DMN may play a role in the positive adaptation of individuals facing numerous adverse events.

In a review of the literature examining the neurobiological underpinnings of resilience in adults, Bolsinger et al. (2018) discovered that individuals with high levels of resilience exhibited larger volumes in the ventromedial prefrontal cortex (vmPFC), the anterior cingulate cortex, and, a lesser extent, the hippocampus, compared to those with low levels of resilience (Bolsinger et al., 2018). Additionally, reduced functional connectivity between the amygdala and the salience network and within the default mode network was associated with increased resilience. Bolsinger et al. also observed that resilience was connected to an enhanced capacity to engage the prefrontal cortex (PFC), leading to more effective regulation of the amygdala through top-down control mechanisms.

Iadipaolo et al. (2018) specifically investigated trait resilience among children and adolescents facing risks such as low socioeconomic status and frequent exposure to adversity. They utilized resting-state functional connectivity analysis. The findings indicated that individuals with high levels of trait resilience spent less time in a dynamic state characterized by positive connectivity between the anterior default mode network and the right central executive network. This particular connectivity pattern is believed to signify enhanced control over the spontaneous processing of internal stimuli, including autobiographical memory recall. It potentially underlies rumination and the tendency to focus on negative thoughts associated with symptoms of depression. These results imply that individuals with high trait resilience engage in less rumination, thus showcasing their resilience against depression. Therefore, the current finding of differences in the DMN connectivity between the two subgroups of individuals with high vs. low resilience aligns with these prior studies.

### Study limitations

Several noteworthy issues should be addressed in future studies. Firstly, while the BBTS offers a valuable framework for participants to reflect upon and report a wide range of experiences, there are inherent limitations associated with relying solely on self-report data. For example, asking participants to retrospectively recall their experienced adversity may lead to underestimation due to forgetting. Moreover, the potential for recall bias or repression of traumatic events in survey responses poses a challenge in accurately gauging the true extent of adversity participants face, particularly in cases where adversities are severe or have occurred in the distant past. Indeed, the inherent nature of self-report surveys limits our ability to capture the complex interplay of feelings, memories, and interpretations associated with each adverse experience. This uncertainty arises as we cannot be certain whether a person is fully conscious of adverse events. However, despite these challenges, the BBTS provides participants with insights into their perception of events, which is fundamental in understanding the subjective impact of adversity. Secondly, in this study, we quantified the number of adversity factors by assigning a value of 1 for the presence of each type of adverse event, and 0 otherwise. However, it is also important to consider the dose–response function for the frequency, duration, and severity of each type of adverse event in future research. Yet, a prior study by You and colleagues (You et al., 2019) has demonstrated that the number of childhood adverse events exerts a stronger influence on certain health outcomes, such as chronic pain conditions and headaches, irrespective of the specific types of trauma experienced. This suggests that the accumulation of adverse experiences may play a more critical role in shaping individual outcomes than the particular nature of each adversity. Thirdly, as aforementioned, our current approach primarily relied on quantitative measures (e.g., based on BBTS), which, although effective in delineating the number of adversities, may not fully encapsulate the depth and subjective experience of each adverse event. However, we recognize that no tool can perfectly capture all repressed experiences, the pivotal concept of the current study centers around giving greater weight to participants’ self-awareness of adverse events, rather than relying solely on the objective quantification of those events. Acknowledging the limitations in capturing the qualitative aspects of adversity is essential. Future research should consider incorporating qualitative methods, such as in-depth interviews or psychological assessments, to capture adversity’s complex and subjective nature. This would enable a more comprehensive understanding of how different adversity factors qualitatively impact individuals’ well-being. Fourthly, some subgroups involved a smaller sample size for the resilience subgroup’s comparisons, which may need more participants in future studies to generalize our current findings. Finally, as this study was cross-sectional in nature, it would be valuable to conduct future longitudinal studies to examine the changes in subjective well-being within individuals following the experience of adverse events. This would provide a more comprehensive understanding of the long-term effects of adversity on subjective well-being.

## Conclusion

From our findings above, we propose the potential mechanism underlying psychological resilience moderates the impact of cumulative adversity on brain regions and subjective well-being. High psychological resilience acts as a protective factor against the negative impact of cumulative adversity on brain regions and subjective well-being. Resilience may help individuals cope more effectively with stressors and challenges, preventing or mitigating the detrimental effects of adversity on brain structure and function. Specifically, resilient individuals may exhibit adaptive neurobiological responses that enable them to maintain emotional regulation, cognitive flexibility, and memory functions mediated by the hippocampus, amygdala, and prefrontal Cortex. Psychological resilience plays a crucial role in buffering the negative impact of cumulative adversity on brain regions and subjective well-being, with implications for understanding stress resilience and well-being interventions.

Additionally, findings from this study add knowledge and highlight the role of psychological resilience in enhancing subjective well-being. Hence, there are implications to clinical practice, i.e., mindfulness training has been suggested as a practice in improving psychological resilience and partially mediates the association between trauma exposures and subjective well-being (Kachadourian et al., 2021). In addition, greater psychological adjustment after trauma has been suggested as a linkage between the presence of mindfulness and acceptance as traits, whereas higher severity of PTSD symptoms and related psychopathology are associated with experiential avoidance, persistent dissociation, and coping strategies involving emotional disengagement (Thompson et al., 2011). Psychosocial interventions that strengthen participants’ resilience can be developed to minimize perceived stress and enhance subjective well-being.

## Data availability statement

The original contributions presented in the study are included in the article/Supplementary material, further inquiries can be directed to the corresponding author/s.

## Ethics statement

The studies involving humans were approved by the Research Ethics Committee (REC) at National Cheng Kung University (NCKU No. 109–419) and the Institute of Review Board (IRB, JA-109-95) of Jen-Ai Hospital. The studies were conducted in accordance with the local legislation and institutional requirements. The participants provided their written informed consent to participate in this study. Written informed consent was obtained from the individual(s) for the publication of any potentially identifiable images or data included in this article.

## Author contributions

SH: Conceptualization, Data curation, Formal analysis, Funding acquisition, Investigation, Methodology, Project administration, Resources, Software, Supervision, Validation, Visualization, Writing – original draft, Writing – review & editing. Y-HC: Funding acquisition, Supervision, Validation, Visualization, Writing – review & editing. Z-FY: Conceptualization, Formal analysis, Methodology, Supervision, Validation, Visualization, Writing – original draft, Writing – review & editing. M-HY: Data curation, Formal analysis, Investigation, Methodology, Resources, Software, Validation, Visualization, Writing – original draft, Writing – review & editing. C-TY: Funding acquisition, Supervision, Validation, Visualization, Writing – review & editing.

## Footnotes

^1^http://surfer.nmr.mgh.harvard.edu/

^2^https://surfer.nmr.mgh.harvard.edu/fswiki/CorticalParcellation

^3^www.nitrc.org/projects/conn

^4^http://www.fil.ion.ucl.ac.uk/spm
