# Supplementary material for: The effect of age and resilience on the dose–response function between the number of adversity factors and subjective well-being
Source: Front Psychol. 2024 Feb 9;15:1332124. doi: 10.3389/fpsyg.2024.1332124 (PMC10884289; doi:10.3389/fpsyg.2024.1332124)
Supplement: Supplementary file 1 [file Data_Sheet_1.docx]

**The effect of age and resilience on the dose-response function between the number of adversity factors and subjective well-being**


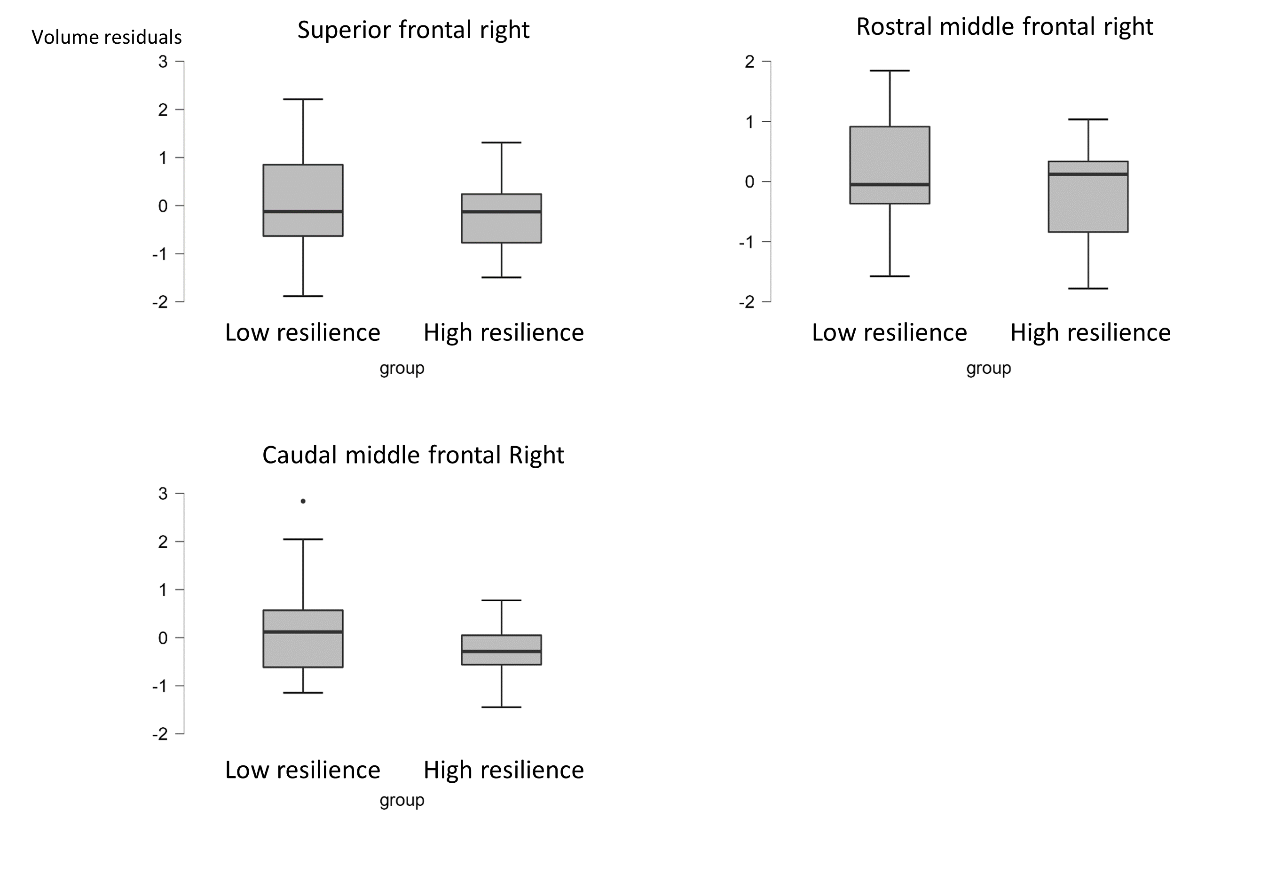


**Figure S1.** Box plot of brain gray matter volume residuals for two subgroups within the high number of adversity factors experienced by participants.


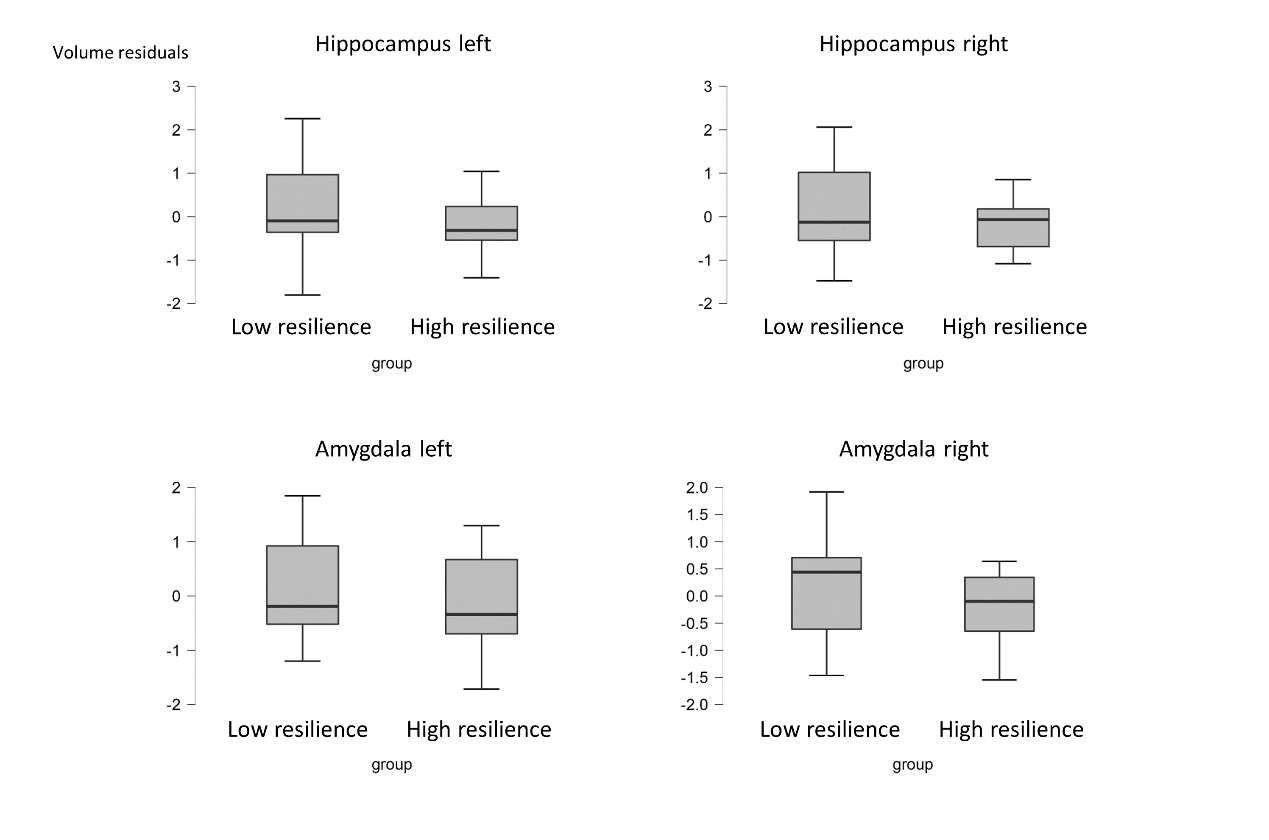


**Figure S2.** Box plot of subcortical volume residuals for two subgroups within the high number of adversity factors experienced by participants.
